# Protein Phosphatases MoPtc5, MoPtc1, and MoPtc2 Contribute to the Vegetative Growth, Stress Adaptation, and Virulence of *Magnaporthe oryzae*

**DOI:** 10.3390/jof11030231

**Published:** 2025-03-18

**Authors:** Jules Biregeya, Frankline Jagero Otieno, Meilian Chen, Anjago Wilfred Mabeche, Abah Felix, Nsanzinshuti Aimable, Yakubu Saddeeq Abubakar, Osakina Aron, Guodong Lu, Zonghua Wang, Yonghe Hong, Wei Tang

**Affiliations:** 1 Fujian Universities Key Laboratory for Plant-Microbe Interaction, College of Life Sciences, Fujian Agriculture and Forestry University, Fuzhou 350002, China; biregeyakayihura2020@gmail.com (J.B.); frank12111@gmail.com (F.J.O.); mabechewilfred@gmail.com (A.W.M.); fabah11@gmail.com (A.F.); nsanziaima@gmail.com (N.A.); ay.saddeeq@yahoo.com (Y.S.A.); aron@wustl.edu (O.A.); lgd@fafu.edu.cn (G.L.); wangzh@fafu.edu.cn (Z.W.); 2 Rice Research Institute, Fujian Academy of Agricultural Sciences, Fuzhou 350018, China; 3 Fuzhou Institute of Oceanography, Minjiang University, Fuzhou 350108, China; meilian2019@mju.edu.cn

**Keywords:** *Magnaporthe oryzae*, synergistic, stress tolerance, protein phosphatases, pathogenesis

## Abstract

Protein phosphatases are crucial enzymes that regulate key cellular processes such as the cell cycle, gene transcription, and translation in eukaryotes. Seven PP2C protein phosphatases have been identified in *Magnaporthe oryzae*. However, their synergistic roles in the pathology and physiology of *M. oryzae* remain poorly investigated. By qRT-PCR analysis, we found that PTC1 and PTC2 are significantly upregulated in the PTC5 deletion mutant. The double deletion of the *MoPTC5*/*MoPTC1* and *MoPTC5*/*MoPTC2* genes significantly reduced hyphal growth, conidiophore formation, sporulation, and virulence in *M. oryzae*. In addition, the double-knockout mutants were increasingly sensitive to different osmotic, oxidative, and cell wall stresses. Western blot analysis revealed that MoPtc5 plays a synergistic function with MoPtc1 and MoPtc2 in the regulation of MoMps1 and MoOsm1 phosphorylation levels. Lastly, appressorium formation and turgor generation were remarkably affected in the Δ*Moptc5*Δ*Moptc1* and Δ*Moptc5*Δ*Moptc2* double-deletion mutants. These findings demonstrate the overlapping roles of PP2c protein phosphatase in the fungal development and pathogenesis of *M. oryzae*.

## 1. Introduction

*Magnaporthe oryzae* is a hemibiotrophic pathogen that threatens rice, wheat, and other cereals globally, causing a devastating blast disease that results in significant annual yield losses [1]. Early reports also demonstrated that rice blast fungus could infect some additional grass species, including *Leersia*, *Pinicum*, *Setaria*, and *Lolium* species [2]. *M. oryzae* is accepted and recognized as a model organism for studying fungal and host interacting pathways [3]. Therefore, the availability of full genomic sequences of pathotypes and that of their hosts has made it more convenient to study fungal genomics [4]. *M. oryzae* infects the host via a structure known as appressoria [5]. This structure accumulates turgor pressure, enabling the generation of mechanical force to penetrate plant cuticles using a penetration peg and release effectors into the plant tissues [6,7,8]. Several signaling pathways regulated by protein kinases and phosphatases have been shown to play a crucial role in appressorium formation in *M. oryzae* [9]. To manage and mitigate the severity of rice blast disease, integrated strategies have been implemented for durable crop resistance, including cultural practices, chemical control, and functional genomic analysis of host–pathogen interaction [10,11,12].

Protein phosphatases are very important components of cellular signaling pathways that regulate fungal development [13]. Type 2C protein phosphatases (PP2C) are subdivided into two classes, namely and Mg^2+^-dependent protein phosphatases [14,15]. The functions of PP2C, including Ptc1, Ptc2, Ptc5, Ptc6, and Ptc7, have been discussed extensively in *Saccharomyces cerevisiae* [16]. Previous research indicated that Ptc2 is primarily involved in the Hog pathway, which is a mitogen-activated protein kinase pathway that regulates stress response mainly related to hyperosmotic stress [17]. Further studies also revealed that Ptc1 is involved in cell wall maintenance, conidiation, RNA splicing, and arrangement of subcellular organelles in filamentous fungi [18,19,20]. Ptc2 and Ptc1 play critical roles in dephosphorylating cyclin-dependent kinases, essential for the regulation of double-strand DNA breakdown by dephosphorylating Rad53 protein kinase [21,22]. In *M. oryzae*, mutants lacking both *MoPTC1* and *MoPTC2* exhibit defects in vegetative growth, virulence, protein phosphorylation, multistresses, and conidiogenesis [23,24]. Additionally, a study in *Aspergillus flavus* demonstrated that the dephosphorylation of PGK1 via Ptc1 and Ptc2 regulates aflatoxin levels and autophagy assays [25]. However, a single deletion of *CaPTC5* in *C. albicans* did not affect tolerance toward various stress agents, including azoles [26]. Additionally, the double knockout of *PTC5* along with other type 2C protein phosphatase genes in *S. cerevisiae* revealed that PP2Cs are important in responses to diverse abiotic stressors [27]. Recently, we revealed that *PTC5* and *PTC7* are important for stress maintenance, asexual reproduction, phosphorylation levels of MoOsm1 and MoMps1, and virulence in *M. oryzae* [28].

Type 2C protein phosphatases have been indicated to play overlapping and indispensable functions in filamentous fungi [27,29]. We applied a molecular biology approach to explore the synergistic roles of *MoPTC5* along with *MoPTC1* or *MoPTC2* in *M. oryzae.* Our study provided evidence that double mutation of *MoPTC5* with *MoPTC1* or *MoPTC2* synergistically altered hyphal growth, fungal sporulation, cell wall integrity, the regulation of MoMps1 and MoOsm1 phosphorylation, and weakened the virulence in *M. oryzae.*

## 2. Materials and Methods

### 2.1. Genes Knockout and Complementation Assays

The split marker assay was employed to knock out *MoPTC5* in *M. oryzae*. The upstream region (A fragment) and downstream region (B fragment) of the genes of interest were amplified. These fragments were then fused with hygromycin fragments (HA and HB) by using overlap extension polymerase chain reaction (SOE PCR), a fragment corresponding to the hygromycin upstream region (HA) and a B fragment ligated to the hygromycin downstream region (HB). The ligated vectors were transformed into Guy11 (wild-type) protoplasts as presented in the early report [30,31]. The grown transformants were screened and confirmed by PCR. To obtain ∆*Moptc5*∆*Moptc1* and ∆*Moptc5*∆*Moptc2* double-mutant constructs, flanking fragments A and B of MoPtc1 and MoPtc2 were amplified and ligated with the Neomycin resistant gene (NE and EO). These ligated constructs were then transformed into ∆*Moptc5* mutant protoplasts, and positive transformants were selected on terrific broth 3 growing media (TB3) containing neomycin and colonies were verified by PCR and Southern blot assay. All primers used in this study are listed in Supplementary Table S1.

### 2.2. Fungal Strains, Media and Culture Conditions

The *M. oryzae* Guy11 was used in this study as a wild-type strain. Complete medium, starch yeast medium, and rice bran medium were utilized for culturing fungi. The hyphae morphology and growth assays were conducted as described in [32]. To discuss the influence of cell wall, oxidative, and osmotic chemicals on mutants and wild-type Guy11, the strains were cultured on CM media containing 200 µg/mL Congo red, 200 µg/mL CFW, 0.01% SDS,1 M of sodium chloride, 1 M of potassium chloride, and 10 mM H_2_O_2_, respectively. The strains were kept in an incubator at 28 °C, and the measurement of mycelial growth, and pictures was performed after ten days post-inoculation. To ascertain the sporulation rate, mycelia plugs were grown on rice bran media at 28 °C; then, after ten days, hyphae were scratched, and plates were exposed to the light for three days to promote conidia formation. Lastly, conidia were counted using light microscopy.

### 2.3. Pathogenicity, Host Penetration, and Analysis of Appressoria Turgor Pressure

For pathogenicity, fungi were grown on solid rice bran media within ten days at 28 °C; later, the spores were collected, and the conidia suspension was adjusted to 5 × 10^4^ spores/mL and sprayed onto the three-week rice seedlings (CO-39). The seedlings were incubated in the darkness for 24 h at 28 °C, then transferred to a growth chamber with a 12 h photoperiod, and disease signs were monitored and examined after 7 days. To investigate the host penetration potential of the mutants, 10 μL of conidial (5 × 10^4^ spores/mL) from the mutants and Guy11 was inoculated on barley leaves. The hyphae penetration rates were checked at 30, 48, and 60 h post-inoculation. A Nikon microscope (Nikon, Nippon Kogaku kogyo kabushikigaisha optical industries Co., Ltd., Tokyo, Japan) was used to view hyphal growth in fungal epidermal cells [33,34]. Incipient cytorrhysis (Appressoria collapsed due to the loss of internal hydraulic turgor pressure) analyses were carried out to investigate the generation of the appressorium turgor pressure in ∆*Moptc5*, ∆*Moptc*5∆*Moptc1*, ∆*Mopt5*∆*Moptc2*, ∆*Moptc5_C*, and wild-type strains. Conidia suspensions were dropped on hydrophobic coverslips (Fisher Scientific, Pittsburgh, PA 15275, USA), and within 24 h, conidia were mixed with glycerol solutions 1 M, 2 M, and 3 M, respectively. Incipient cytorrhysis and glycogen mobilization were monitored under a microscope.

### 2.4. RNA Extraction, Quantitative Real-Time PCR (qRT-PCR), and RT-PCR Assays

The mutants and wild-type strains (Guy11, ∆*Moptc5*, ∆*Moptc5*∆*Moptc1*, and ∆*Moptc5*∆*Moptc2*) were inoculated in liquid CM, and shaken in a machine at 110 rpm, 28 °C, for a 3-day period. Mycelia were collected, washed with ddH_2_O, dried, and crushed into fine powder by using a mortar in the nitrogen solution. The extraction of total RNA from each strain was performed following the protocol of an Eastep TM extraction kit (Promega, Beijing Biotech Co., Ltd., Beijing, China). Quantitative real-time PCR (qRT-PCR) was conducted following Promega Super Real Premix kit instructions. The qRT-PCR was performed in an Eppendorf Realplex2 master cycler (Eppendorf China Ltd., Shanghai, China), and analysis was performed using (2^−ΔΔCT^) as previously reported by [35].

### 2.5. Southern Blotting Assay

For the Southern blotting, we first extracted fungal genomes from fungal strains using the cetytrimethylammonium bromide method (CTAB method). The digestion of the genomic DNA was carried out by using specific restriction enzymes PstI and ClaI, respectively (New England Biolab Co., Ltd. Beijing, China). The digested DNA product was separated by running a gel electrophoresis assay. The gel was later moved to a specific membrane (Merck) that was positively charged. Nucleic acid probes with sequences complementary to the region of targeted fragments were used for hybridization. During hybridization, the labeled probes were incubated overnight, together with the DNA, in a hybridizer machine at 42 °C to promote the hybridization of the complementary sequences. Unhybridized probes were removed by washing the membrane with 2× SSC+ SDS and with maleic acid + tween 20 solutions. A detection starter kit I (Merck KGa, Darmstadt, Germany) and images were captured using Tanon 5200 chemiluminescent imaging machine (Tanon Science &Technology Co., Ltd., Shanghai, China) (Supplementary Figure S1).

### 2.6. Extraction of Proteins and Western Blotting Assays

The fungal mycelia plugs were cultured in the liquid CM media and shaken for 2–3 days at 28 °C. After that, mycelia were collected and crushed into powder by using a mortar, and then, 1–2 g of the mycelial powder was resuspended in 1 mL of protein lysis mixed with 10 µL of phenylmethyl sulphonyl fluoride (PMSF) and 10 µL of a proteinase inhibitor. The mixed solution was incubated in ice for a half hour and vortexed by inverting the tubes every 10 min, and afterward, the samples were centrifuged for 20 min at 4 °C. The supernatants were sucked and added into 2 mL tubes; lastly, sodium dodecyl sulfate buffer (SDS buffer) was added before storage at −21 °C for future use. The Western blot assay, SDS -PAGE (10% polyacrylamide) assay, was used for the separation of proteins. The targeted proteins were detected using the primary antibodies (P-p38 MAPK, and P44/42MAPK), along with conjugated secondary antibodies (Goat antirabbit& mouse IgG-HRP), and an anti-beta actin mab was utilized as a control antibody. Finally, the protein phosphorylation signals were detected following the instructions of a Western blotting Kit (Epizyme, SQ201, Shanghai, China), and photographs were taken by Imaging System (Tanon Science &Technology Co., Ltd., Shanghai, China).

### 2.7. Microscopic Examination Assays

Conidiophore development, conidia formation, appressorium formation, invasive hyphae, conidia germination on the hydrophobic slides, glycogen mobilization in appressoria, and appressorial turgor generation were viewed under a Nikon TiE system (Optical Industries Co., Ltd, Tokyo, Japan). To observe the cell wall thickness of the indicated strains, we inoculated them in liquid CM at 28 °C for 3 days. Mycelia were then harvested, and transmission electron micrographs of the transverse sections of the cell walls were obtained using transmission electron microscopy (TEM).

### 2.8. Statistical Analysis

Graphpad Prism version 8 was used for statistical analysis. The data from biological replicates were analyzed using one-way ANOVA (including nonparametric tests). ImageJ software (ImageJ 1.53e/Java) was utilized for the densitometric analysis of the Western blot bands.

## 3. Results

### 3.1. Generation of ∆Moptc5∆Moptc1 and ∆Moptc5∆Moptc2 Deletion Mutants

To establish the synergistic roles of MoPtc5 with the MoPtc1 and MoPtc2 genes in the development and pathogenicity of the rice blast fungus, we deleted both genes in the background of ∆*Moptc5*. The target gene replacement vector was constructed by amplifying the upstream and downstream regions of *MoPTC1* and *MoPTC2*. These regions were then ligated with split parts of the Neomycin-resistant gene Open Reading Frame (NE and EO) by using gene splicing by overlap extension PCR (SOE-PCR) and transform mutant protoplasts. In this case, the Neomycin antibiotic was added as a selection marker at ratios of 1:3 and 3:6 to the first and second layers of the TB3 medium, respectively. The neomycin-resistant transformants were screened by PCR using ORF and UAH primers. Further confirmation was conducted by the Southern blotting method. NEO—Full length of the Neomycin-resistance gene; NE-5′—part of the Neomycin-resistance gene; EO-3′—part of the Neomycin-resistance gene (Figure S1).

### 3.2. Phylogenetic Analyses and Identification of MoPtc1, MoPtc2, and MoPtc5

**Figure 1:**
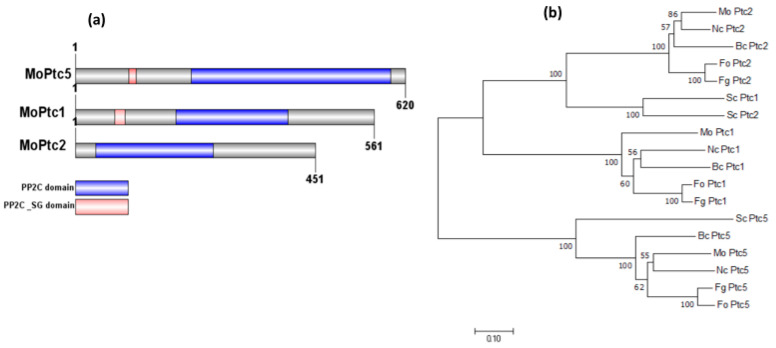
Domain architectures and phylogenetic analyses of MoPtc1, MoPtc2, and MoPtc5 proteins in *M. oryzae*. (**a**) Prediction of functional domains with the SMART database and scheme produced by IBS software 2.0. (**b**) Phylogenetic analysis of Ptc5, Ptc1, and Ptc2 from several filamentous fungi, including *Nc*: *Neurospora crassa*;* Bc*:* Botrytis cinerea*;* Fo*:* Fusarium oxysporum*;* Fg*:* Fusarium graminearum*; *Sc*: *Saccharomyces cerevisiae*; and *Mo*:* Magnaporthe oryzae*.

The homologs of *PTC5*, *PTC1*, and *PTC2* in blast fungus were identified from the fungidb website http://fungidb.org/fungidb/ (accessed on 3 March 2024) via BlastP search, based on the amino acid sequences from *S. cerevisiae*, and were termed *MoPTC5* (*MGG_03154*), *MoPTC1* (*MGG_05207*), and *MoPTC2* (*MGG_01351*), respectively. We identified the PTC1, PTC2, and PTC5 homologs in other fungi via NCBI (National Center for Biotechnology Information) blast. Domain annotation of the proteins was generated using IBS software 2.0, while MEGAx software 10.2.6 was used to build the phylogenetic tree with bootstrap analysis [36]. The analysis demonstrated that MoPtc5, MoPtc1, and MoPtc2 possess two conserved domains (PP2C domain and PP2C_SG domain) (Figure 1a). Additionally, the phylogenetic assay indicated that MoPtc1 and MoPtc2 are most closely related to their orthologs in *Neurospora crassa*, while MoPtc5 has the closest homology to *N. crassa* and *Botrytis cinera* (Figure 1b).

### 3.3. ∆Moptc5∆Moptc1 and ∆Moptc5∆Moptc2 Strongly Affect the Hyphal Growth and Sporulation of M. oryzae

**Figure 2:**
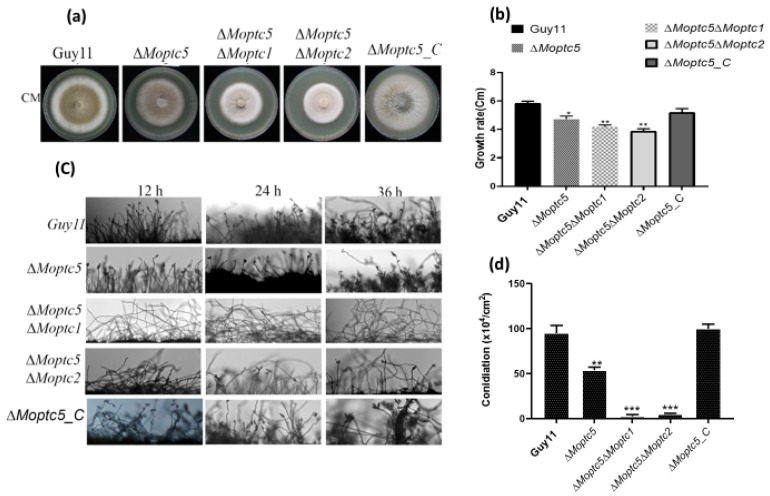
Functional redundancy of *MoPTC5* with *MoPTC1* or *MoPTC2* in the development of *M. oryzae*. (**a**,**b**) Growth assay of Guy11, ∆*Moptc5*, ∆*Moptc5*∆*Moptc1*, and ∆*Moptc5*∆*Moptc2* mutants. (**c**) Conidiophore-producing ability of the mutants compared to Guy11. (**d**) Conidiation rate of mutant and Guy11 grown on rice bran media for ten days. The error bars displayed the standard mean from three biological repeats, asterisks indicate significant differences, and data analysis was performed by using one-way ANOVA with multiple comparison tests in the Graph Prism 8 software). Asterisks reveal the significances (* *p* < 0.05; ** *p* < 0.01, and *** *p* < 0.001).

We initially conducted qRT-PCR and observed significant up-regulation of *MoPTC2* and *MoPTC1* in the *MoPTC5* mutant (Supplementary Figure S2), suggesting the redundancy of *MoPTC5*, *MoPTC1*, and *MoPTC2* in *M. oryzae*. Subsequently, double gene knockout of *MoPTC5* with either *MoPTC1* or *MoPTC2* was generated in the ∆*Moptc5* mutant by replacing the ORF (Open reading flame) of *MoPTC1* or *MoPTC2* with a neomycin resistance gene cassette. Culturing wild-type strain Guy11 and the mutant strains ∆*Moptc5*, ∆*Moptc5*∆*Moptc1*, and ∆*Moptc5*∆*Moptc2* along with the complemented strain on CM revealed significantly reduced hyphae growth of the ∆*Moptc5*∆*Moptc1* and ∆*Moptc5*∆*Moptc2* double mutants compared to ∆*Moptc5* and Guy11 (Figure 2a,b). Conidiation was remarkably reduced in ∆*Moptc5*∆*Moptc1* and remarkably reduced in ∆*Moptc5*∆*Moptc2* in comparison to ∆*Moptc5* and the wild type cultured on solid rice bran media (RBM) (Figure 2d). Consistent with this result, ∆*Moptc5*∆*Moptc1* and ∆*Moptc5*∆*Moptc2* failed to produce conidiophores at diverse experimental time points of 12, 24, and 36 h (Figure 2c). Overall, the findings suggest the crucial role of *MoPTC5* with *MoPTC1* or *MoPTC2* in the hyphal growth and asexual reproduction of blast fungus.

### 3.4. MoPTC5, MoPTC1, and MoPTC2 in Cell Wall Maintenance and Mps1 Phosphorylation Level

**Figure 3:**
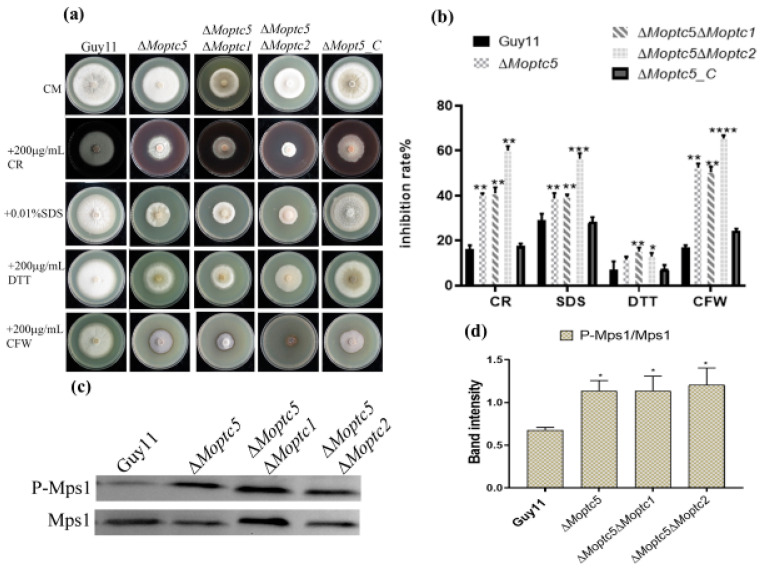
Responses of the ∆*Moptc5*∆*Moptc1* and ∆*Moptc5*∆*Moptc2* mutants toward diverse cell wall stressors. (**a**) Response of Guy11, ∆*Moptc5*, ∆*Moptc5*∆*Moptc1*, and ∆*Moptc5*∆*Moptc2* strains cultured on the SDS, CR, CFW, and DTT. (**b**) Analysis of the inhibition rates of the individual strains due to the influence of the cell-wall-stress-inducing agents. (**c**,**d**) Western blotting analysis of Mps1 phosphorylation in ∆*Moptc5*, ∆*Moptc5*∆*Moptc1*, and ∆*Moptc5*∆*Moptc2* mutants in comparison to the wild type (Guy11). The error bars demonstrate the mean standard error from experimental replicates. Asterisks display the significance difference (* *p* < 0.05; ** *p* < 0.01; *** *p* < 0.001; **** *p* < 0.0001); one-way ANOVA was used to analyze the data.

To further investigate the roles of MoPtc5 in conjunction with MoPtc1 or MoPtc2 in regulating the cell wall maintenance of *M. oryzae*, we analyzed the growth inhibition rate of mutants grown on CM containing different stress chemicals, including DTT, SDS, CFW, and CR, for ten days of incubation at 28 °C. The results showed that the growth of ∆*Moptc5*, ∆*Moptc5*∆*Moptc1*, and ∆*Moptc5*∆*Moptc2* on CM supplemented with SDS, CR, CFW, and DTT was significantly inhibited. Especially, ∆*Moptc5*∆*Moptc2* displayed a higher sensitivity response compared to other mutants when spotted on CM supplemented with SDS, CR, and CFW (Figure 3a,b). We then performed Western blotting to evaluate the activation of MoMps1 in the mutants. The results indicated an elevation of the phosphorylation rater of MoMps1 protein in ∆*Moptc5*, ∆*Moptc5*∆*Moptc1*, and ∆*Moptc5*∆*Moptc2* strains (Figure 3c,d). The findings demonstrate that *MoPTC5*/*MoPTC1* and *MoPTC5*/*MoPTC2* are collaboratively involved in the integrity of the cell wall and the phosphorylation of MoMps1 in *M. oryzae*.

### 3.5. Double Knockout of MoPTC5 with MoPTC1 and MoPTC2 Did Not Influence the Cell Wall Thickness of Rice Blast Fungus

**Figure 4:**
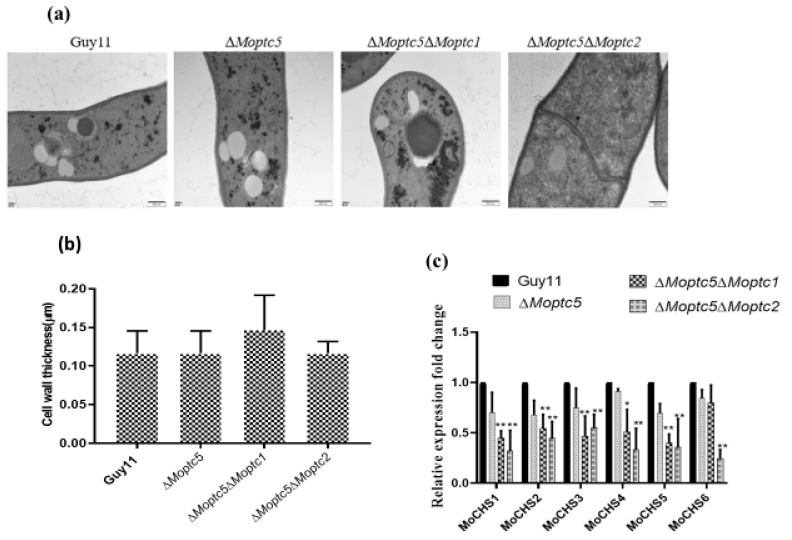
Double deletion of *MoPTC5* with *MoPTC1 and MoPTC2* did not alter cell wall size or thickness. (**a**) Observations of cell wall thickness in hyphae harvested from Guy11, Δ*Moptc5*, Δ*Moptc5*Δ*Moptc1*, and Δ*Moptc5*Δ*Moptc* mutants via transmission electron microscopy. (**b**) Bar graphs presenting mycelial cell wall thickness for the WT strain and mutants. (**c**) Expression levels of genes encoding for chitin synthase. The actin gene was selected and used as a housekeeping gene. Data are from three biological replicates. Error bars indicate standard deviations, and asterisks demonstrate significant differences. * *p* < 0.05; ** *p* < 0.01. Scale bar = 200 nm.

The cell wall serves as a robust protection safeguarding the plasma membrane and intracellular contents of plants and many microbes [37]. To investigate the potential involvement of MoPtc5 along with MoPtc1 or MoPtc2 in the cell wall thickness of the blast fungus, we cultured strains on CM for three days. The fungal hyphae were then harvested, washed with ddH_2_O, dried with clean filter papers, and preserved in phosphate buffer. Transverse sections of hyphal cell walls were imaged using electron transmission microscopy (TEM). However, we observed no discernible difference in the thickness of cell walls among the wild-type strains and Δ*Moptc5*, Δ*Moptc5*Δ*Moptc1*, and Δ*Moptc5*Δ*Moptc2* mutants (Figure 4a,b). Chitin represents a vital constituent of fungal cell walls. Hence, we conducted further analysis on the transcription levels of chitin synthases in *M. oryzae* through qRT-PCR to gain a clearer understanding of how these proteins regulate cell wall stress. Our results showed a remarkably decreased expression of the chitin synthase-encoding genes in the mutants compared to the wild type (Figure 4c).

### 3.6. Moptc5, Along with Moptc1 and Moptc2, Plays Important Roles in Osmotic, Oxidative Stress, and MoOsm1 Phosphorylation in M. oryzae

**Figure 5:**
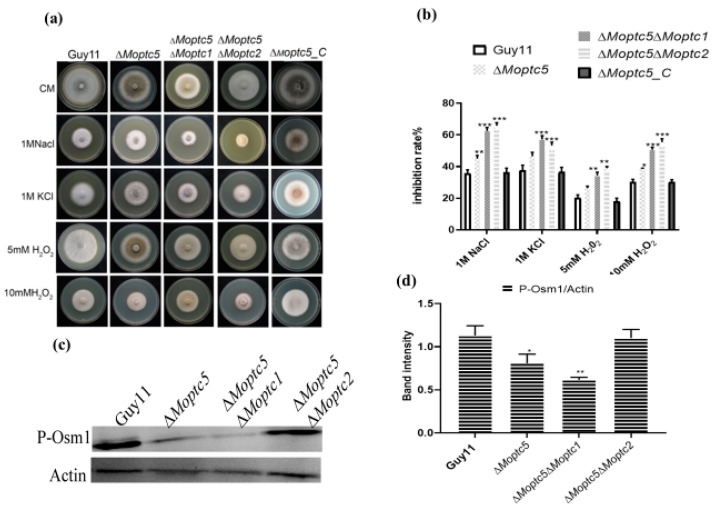
Tolerance of ∆*Moptc5*∆*Moptc1 and* ∆*Moptc5*∆*Moptc2* mutants to multiple hyperosmotic and oxidative stresses. (**a**) Mycelial growth of Guy11, ∆*Moptc5*, ∆*Moptc5*∆*Moptc1*, and ∆*Moptc5*∆*Moptc2* strains on CM containing oxidative and osmotic chemicals. (**b**) Calculation of the inhibition rate of the mutants compared to Guy11 strains under the influence of osmotic and oxidative stresses. (**c**,**d**) Evaluation of MoOsm1 phosphorylation in Guy11, ∆*Moptc5*, ∆*Moptc5*∆*Moptc1*, and ∆*Moptc5*∆*Moptc2* mutants. Error bars represent the mean from replicates; one-way ANOVA was used for data analysis with multiple comparison tests in Graph Prism 8. * *p* < 0.05; ** *p* < 0.01; *** *p* < 0.001.

To determine the functional redundancy of *MoPTC5*, *MoPTC1*, and *MoPTC2* in *M. oryzae* under oxidative and hyperosmotic stresses, we cultured Guy11, Δ*Moptc5*, Δ*Moptc5*Δ*Moptc1*, and Δ*Moptc5*Δ*Moptc2* on CM supplemented with osmotic and oxidative stressors chemicals including potassium chloride (1 M KCl), sodium chloride (1 M NaCl), 5 mM H_2_O_2_, and 10 mM H_2_O_2_. Growth measurements were recorded after ten days post-incubation. Our findings demonstrate that both Δ*Moptc5*Δ*Moptc1* and Δ*Moptc5*Δ*Moptc2* exhibited higher sensitivity to hyperosmotic and oxidative stress induced by 1 M NaCl, 1 M KCl, 5 mM H_2_O_2_, or 10 mM H_2_O_2_ in comparison to the Guy11 and Δ*Moptc5* mutant (Figure 5a,b). A previous study demonstrated that the double deletion of ∆*Moptc5*∆*Moptc7* reduced the MoOsm1 phosphorylation level. Consequently, we conducted a Western blotting assay to quantify the relative abundance of the osmoregulatory protein MoOsm1 in ∆*Moptc5*, ∆*Moptc5*∆*Moptc1*, ∆*Moptc5*∆*Moptc2*, and Guy11. Our results reveal a low abundance of MoOsm1 in ∆*Moptc5* and ∆*Moptc5*∆*Moptc1* mutants but not in Δ*Moptc5Moptc2* (Figure 5c,d). Altogether, we proposed that *MoPtc5*, in conjunction with MoPtc1 and MoPtc2, significantly contributes to the hyperosmotic and oxidative tolerance of *M. oryzae*, as well as MoOsm1 phosphorylation.

### 3.7. ∆Moptc5∆Moptc1 and ∆Moptc5∆Moptc2 Mutants Are Remarkably Delayed in Appressoria Formation

**Figure 6:**
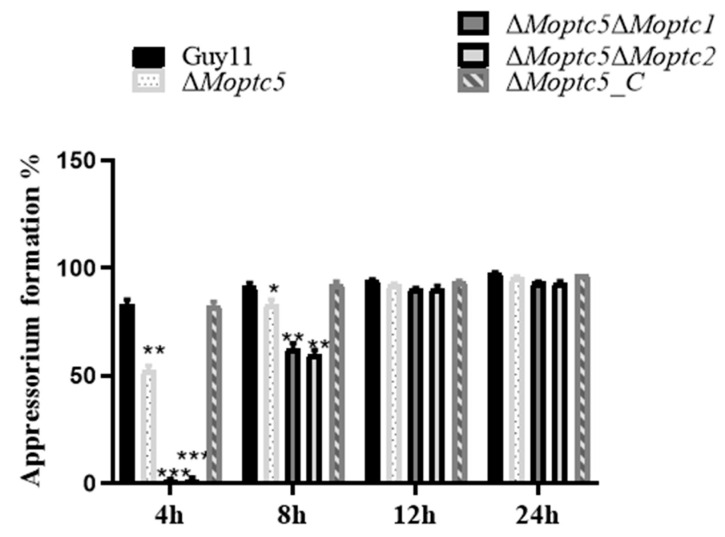
Appressorium formation rate of ∆*Moptc5*, ∆*Moptc5*∆*Moptc1*, and ∆*Moptc5*∆*Moptc2* mutants and wild-type strains on the hydrophobic surface at 4, 8,12, and 24 h post-inoculation. We used one-way ANOVA to analyze the data with multiple comparison tests in Graph Prism 8. Asterisks indicate a significant difference in comparison to the wild type. * *p* < 0.05; ** *p* < 0.01; *** *p* < 0.001.

The appressorium serves as the primary infectious structure facilitating the penetration of rice blast fungus. Therefore, to examine *MoPTC5′*s role alongside *MoPTC1* or *MoPTC2* in appressorium formation, conidial suspensions of ∆*Moptc5*, ∆*Moptc5*∆*Moptc1*, ∆*Moptc5*∆*Moptc2*, and Guy11 (wild type) were initially placed on hydrophobic coverslips. Appressoria formation was checked and viewed under a microscope at 4, 8, 12, and 24 h post-inoculation, respectively. Findings generated from this assay indicated that both double-gene-deletion mutants failed to develop appressorium at 4 h post-inoculation. However, the appressorium formation rate of the ∆*Moptc5*∆*Moptc1* and ∆*Moptc5*∆*Moptc2* mutants gradually increased over time until comparable to Guy11 (Figure 6). Taken together, results demonstrated that the deletion of *MoPTC5*, along with *MoPTC1* or *MoPTC2*, merely delayed the appressorium formation of *M. oryzae*.

### 3.8. MoPTC5, MoPTC1, and MoPTC2 Synergistically Influence Appressoria Turgor Generation and the Utilization of Glycogen in M. oryzae

**Figure 7:**
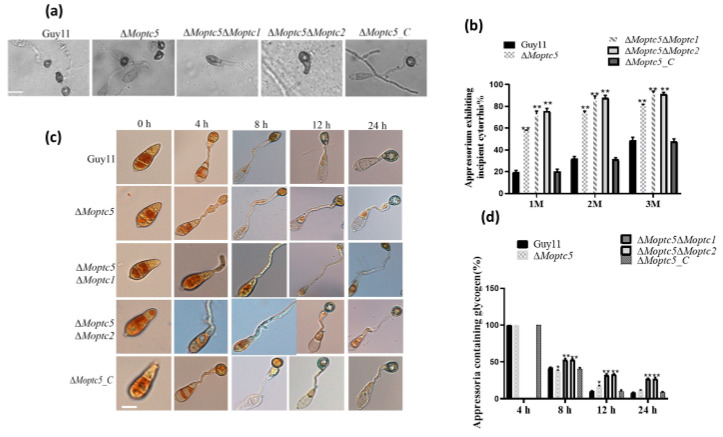
Double gene deletion of *MoPTC5* with either *MoPTC1* or *MoPTC2* resulted in compromised turgor generation and glycogen mobilization during appressorium development in rice blast fungus. (**a**,**b**) Appressoria images and percentages collapsed by treatment with glycerol. These appressoria were treated with different concentrations of glycerol solutions (1 M, 2 M, and 3 M). Collapse of the appressoria was observed and captured under a microscope. (**c**,**d**) illustrates the rate of glycogen mobilization in the indicated strains at 0, 4, 8, 12, and 24 h time points during germination and appressorium formation. One-way ANOVA was used to analyze the data with multiple comparison tests in Graph Prism 8. * *p* < 0.05; ** *p* < 0.01. Scale bar = 20 µm.

To ascertain the synergistic roles of MoPtc5 in tandem with MoPtc1 and MoPtc2 during appressorium penetration in host epidermal cells, the appressorium turgor generation across all mutants and the wild type was examined, respectively. Conidia sourced from the aforementioned strains were inoculated onto hydrophobic coverslips, followed by exposure to varying concentrations of glycerol after 24 h. The percentage of collapsed appressoria was viewed and monitored under a microscope. Our findings demonstrate that a significant number of the appressoriums produced by Δ*Moptc5*Δ*Moptc1* and Δ*Moptc5*Δ*Moptc2* were collapsed under diverse glycerol concentrations (Figure 7a,b). Moreover, the appressorium formation of rice blast fungus necessitates the efficient transfer of nutrients, such as glycogen and lipid droplets, from conidia, acting as essential material sources for turgor generation post-maturation. Effective nutrient transport profoundly influences the virulence of blast fungus. Thus, to assess glycogen mobilization from conidia to appressoria, conidia were harvested and incubated at 28 °C for 2, 4, 8, 12, and 24 h, respectively, and KI/I_2_ was used to stain conidia before being imaged under a microscope. These findings unveiled a delay of glycogen mobilization from conidium to appressoria in the double mutants (Figure 7c,d). In summary, these findings underscore the pivotal contribution of MoPtc5 alongside MoPtc1 and MoPtc2 in appressorium turgor generation, primarily through the regulation of glycogen utilization.

### 3.9. ∆Moptc5∆Moptc1 and ∆Moptc5∆Moptc2 Strains Weakened Virulence to Infect Rice Leaves

**Figure 8:**
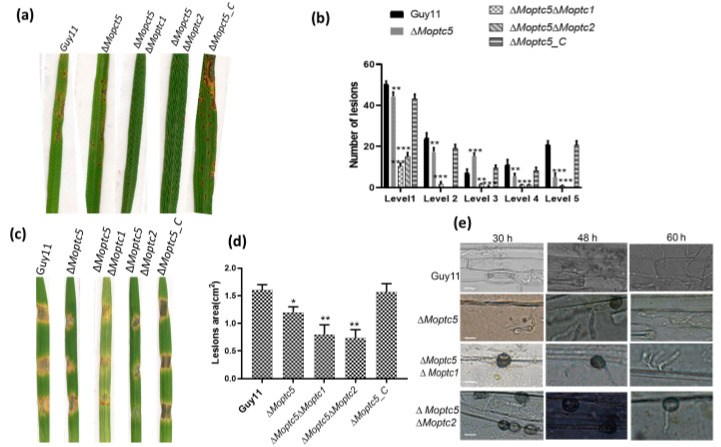
Double deletion of *MoPTC5* with either *MoPTC1* or *MoPTC2* underscores their redundant necessity for virulence in *M. oryzae*. (**a**,**b**) Pathogenicity assay results and the corresponding lesion numbers observed on three-week-old rice seedlings (CO39 cultivar) by Guy11, ∆*Moptc5*, ∆*Moptc5*∆*Moptc1*, and ∆*Moptc5*∆*Moptc2*. (**c**,**d**) The infection assay was conducted on barley leaves using mycelia plugs from Guy11, ∆*Moptc5*, ∆*Moptc5*∆*Moptc1*, and ∆*Moptc5*∆*Moptc2* along with the quantification of the lesion area. (**e**) The development and invasion of hyphae in barley cells at different time points, 12, 24, and 36 h. * *p* < 0.05; ** *p* < 0.01; *** *p* < 0.001. Scale bar = 10 µm.

Protein phosphorylation is an essential mechanism for infection-related morphogenesis [38]. Moreover, the impact of *M. oryzae* protein phosphatase genes on virulence remains inadequately explored. To elucidate the combined effect of double mutations in *MoPTC5* along with *MoPTC1* and *MoPTC2* on fungal virulence, we harvested spores from Guy11, ∆*Moptc*5, ∆*Moptc*5∆*Moptc*1, and ∆*Moptc*5∆*Moptc*2 and sprayed them on the rice seedlings. A striking decrease in pathogenicity was observed in the double mutants in comparison to ∆*Moptc*5 and Guy11 (Figure 8a,b). Barley is an excellent model for *M. oryzae*–plant interaction due to its fast-growing ability [33]. Similarly, a significant reduction in virulence on barley leaves was evident using mycelial plugs (Figure 8c,d). Further analysis revealed a lower penetration rate of hyphae in the cuticle cells of barley at different time points for the double-gene-deletion mutants (Figure 8e). Collectively, our findings demonstrated the collaborative influence of *MoPTC5*, *MoPTC1*, and *MoPTC2* on the virulence of *M. oryzae.*

## 4. Discussion

The main purpose of this research was to reveal the synergistic functions of PP2C MoPtc5 along with MoPtc1 and MoPtc2 in the virulence and development of the rice blast fungus. To achieve this purpose, we initially deleted *MoPTC5* and subsequently knocked out *MoPTC1* and *MoPTC2* in the ∆*Moptc5* mutant. Our investigations indicate that all the mutant strains were viable on different growing mediums. Phylogenetic analysis revealed a close relationship among the proteins MoPtc5, MoPtc1, and MoPtc2, as well as their homologs in other filamentous fungi. This suggests that these proteins have undergone functional differentiation over an extended evolutionary period, which may imply their specialized roles in regulating diverse physiological processes in *M. oryzae*. Understanding these evolutionary relationships can provide insights into the molecular adaptations that have enabled these fungi to thrive in various environments.

Spore formation is critical in filamentous fungi, with spores or conidia developing on vegetative hyphal structures known as conidiophores [39]. Previous studies reported a slight decrease in fungal growth and sporulation in the ∆*Moptc5* single mutant, highlighting the importance of MoPtc5 in these processes [28]. Our findings revealed a very significant reduction in conidiation in the double mutants ∆*Moptc5*∆*Moptc1* and ∆*Moptc5*∆*Moptc2*, underscoring the important role of *MoPTC5*, *MoPTC1*, and *MoPTC2* in sporulation regulation [23,24]. This reduction may be influenced by various environmental factors, such as aeration and low expression levels of conidiation-related genes, which can impact the metabolic and physiological processes occurring during sporulation. The identification of these interactions lays the groundwork for potential biotechnological applications, such as the development of fungal strains with modified sporulation patterns for agricultural use [40,41].

The cell wall is important for cellular integrity and shielding against environmental signals [42]. In our research, we evaluated the impact of diverse cell wall stressor chemicals, revealing higher sensitivity in the double mutant ∆*Moptc5*∆*Moptc2* compared to ∆*Moptc5*, ∆*Moptc5*∆*Moptc1*, and the wild-type strains. This increased sensitivity suggests that the combined loss of MoPtc5 and MoPtc2 severely compromises the cell wall integrity, indicating that these proteins may be part of a larger signaling network that maintains cell wall structure and function [27]. Previous studies indicated a modest sensitivity of ∆*Moptc5* to cell wall stressors, but our findings highlight the more pronounced effects seen in the double mutants, suggesting a critical interplay between these phosphatases in stress response mechanisms [28]. Western blot analysis confirmed that ∆*Moptc5*, ∆*Moptc5*∆*Moptc1*, and ∆*Moptc5*∆*Moptc2* are crucial for the phosphorylation of Mps1, suggesting their involvement in MoMps1 phosphorylation activation in *M. oryzae*. In conclusion, MoPtc5, MoPtc1, and MoPtc*2* synergistically regulate cell wall maintenance and participate in signal transduction pathways essential for cell wall maintenance.

Chitin was reported as an abundant component of fungal and insect cell walls, which protect the organisms against environmental stimuli [43]. In our study, we investigated and measured the cell wall sizes of ∆*Moptc5*, ∆*Moptc5*∆*Moptc1*, and ∆*Moptc5*∆*Moptc2* and the wild-type strains. The results unveiled combined effects of *MoPTC5*, *MoPTC1*, and *MoPTC2* had no direct influence on the fungal cell wall thickness, which is in line with early results [28]. Moreover, we observed the downregulation of chitinase genes in the double mutants, suggesting that *MoPTC5* in conjugation with *MoPTC1* and *MoPTC2* negatively regulates the expression of chitinase encoding genes, possibly through shared transcription factors for these orthologous genes [44].

Additionally, we investigated the synergistic functions of *MoPTC5* with *MoPTC1* and *MoPTC2* in osmotic and oxidative responses. The double mutants ∆*Moptc5*∆*Moptc1* and ∆*Moptc5*∆*Moptc2* showed a higher inhibition rate on media containing 1 M NaCl and 1 M KCl [45,46]. This observation suggests that these protein phosphatases may be essential for co-regulating cellular sensitivity to various osmoregulatory agents, which is crucial for maintaining cellular homeostasis and promoting fungal development under stress conditions. Furthermore, the increased sensitivity of ∆*Moptc5∆Moptc2* to oxidative stress (10 mM H_2_O_2_) suggests that these genes play important functions in the tolerance to oxidative and osmotic stresses [47]. This finding highlights a complex interplay between these phosphatases and stress response pathways, which has not been extensively explored in previous studies. To establish the genetic redundancy of these three protein phosphatases, we also examined the phosphorylation level of the osmoregulatory protein MoOsm1 in the single and double mutants. We found that the MoOsm1 phosphorylation level significantly decreased in ∆*Moptc5* and ∆*Moptc5*∆*Moptc1*. Previous studies demonstrated that MoPtc1 acts as a negative regulator of Osm1 during hypoosmotic stress, while MoPtc2, MoPtc5, and MoPtc7 positively regulate this pathway, indicating functional differentiation over time [24,25]. This implies that MoPtc1 may negatively regulate Osm1 phosphorylation through MoPtc5, while MoPtc2 positively regulates Osm1 phosphorylation, also via MoPtc5. Consequently, the knockout of any *PP2C* gene disrupts the phosphorylation process of Osm1, subsequently affecting the osmotic and oxidative stress responses of the rice blast fungus. This underscores the complementary roles of *MoPTC1*, *MoPTC2*, and *MoPTC5* as integral components in the activation and regulation of osmoregulatory pathways involving glycerol, highlighting the intricate mechanisms by which *M. oryzae* adapts to environmental challenges.

Pathogenicity is crucial in fungi–host interaction. Some factors are important for the establishment of fungal virulence, including conidia attachment, germination on the surface of the host, and its colonization [48]. In this work, we assayed the pathogenicity levels of the various strains on susceptible rice cultivars and barley. Remarkably, we revealed a severe defect in the virulence of the double mutants ∆*Moptc5*∆*Moptc1* and ∆*Moptc5*∆*Moptc2*. Our findings also indicated a high reduction in hyphal penetration and invasion of barley epidermal cells in double mutants. Previous publications showed that the single mutants ∆*Moptc5*, ∆*Moptc1*, and ∆*Moptc2* are required in the pathogenesis of the rice blast fungus [23,49,50]. In this research, we reasoned that PP2C and type 2C protein phosphatases *MoPTC5*, *MoPTC1*, and *MoPTC2* complementarily regulate fungal virulence.

The double mutants ∆*Moptc5*∆*Moptc1* and ∆*Moptc5*∆*Moptc2* are impaired in an appressorium formation at 4 h and 8 h after inoculation on hydrophobic coverslips, compared to the single mutant ∆*Moptc5* and the WT strains. This might be possibly caused by the hyperactivation of the Pmk1 pathway, which has previously been revealed to cause abnormalities in appressorium morphogenesis and pathogenicity [51]. From these results, we suggest that *MoPTC5* in collaboration with *MoPTC1* and *MoPTC2* synergistically regulates appressorium formation and maturation. Appressorium turgor pressure was also examined in various strains. We observed a higher percentage of collapsed appressoria and slowed glycogen mobilization and utilization from conidia to appressoria in ∆*Moptc5*∆*Moptc1*, ∆*Moptc5*∆*Moptc2* than in ∆*Moptc5* and the wild type. However, we are still unclear about the pathways regulating the movement of conidium content to mature appressorium. The findings of incipient cytorrhysis are consistent with the defects of penetration and hyphae invasion in barley epidermal cells, which is also consistent with early studies [52,53].

## 5. Conclusions

Rice blast disease is severe across the world, ultimately leading to losses of 10%–30% for rice, wheat, and other cereals globally. This investigation, carried out on PP2C, the type 2C protein phosphatases, provided evidence of the collaboration of *MoPTC5* with *MoPTC1* and *MoPTC2* in the regulation of sporulation rate, hyphal growth, multi-stress adaptation, and virulence of *M. oryzae* (Table S3). Generally, the findings of this study demonstrate the overlapping roles of type 2C protein phosphatases; in the future, we will identify the substrate proteins and interacting partners of *MoPtc5.* This research revealed new insights into the development and pathogenesis of *M. oryzae*.

## Data Availability

Data are provided within the manuscript or Supplementary Information Files.

## Supplementary Materials

The following supporting information can be downloaded at: https://www.mdpi.com/article/10.3390/jof11030231/s1. Figure S1: Display Southern blots confirmations. (A,B) ∆*Moptc*5∆*Moptc1*. (C,D) ∆*Moptc5*∆*Moptc2* replacements by insertion of hygromycin phosphotransferase gene (Hph) in opening reading frame regions (ORF). Figure S2: Transcripts expression profile of *MoPTC1, MoPTC2, MoPTC6* and *MoPTC7* in Δ*Moptc5* mutant. (β-actin gene was used as reference). Table S1: Fungal strains (wild-type and mutants) employed during this study. Table S2: List of primers used in this study. Table S3: Physiological effects caused by deletion MoPtc5, MoPtc1 and MoPtc2 in *Magnaporthe oryzae*.
