# Supplementary material for: Protein Phosphatases MoPtc5, MoPtc1, and MoPtc2 Contribute to the Vegetative Growth, Stress Adaptation, and Virulence of Magnaporthe oryzae
Source: J Fungi (Basel). 2025 Mar 18;11(3):231. doi: 10.3390/jof11030231 (PMC11943610; doi:10.3390/jof11030231)
Supplement: Supplementary file 1 [file jof-11-00231-s001.zip › jof-3501410-supplementary.pdf]

Supplementary Figures

Journal name : Journal of fungi

Protein phosphatases MoPtc5, MoPtc1, and MoPtc2 contribute to the vegetative growth, stress adaptation, and virulence of *Magnaporthe oryzae*

Jules Biregeya [1,3*], Frankline Jagero Otieno [3*], Meilian Chen [4], Anjago Wilfred Mabeche [1], Abah Felix [3], Nsanzinshuti Aimable [1], Yakubu Saddeeq [3], Osakina Aron [1,3], Guodong Lu [1,3], Zonghua Wang[1,3,4#], Yonghe Hong [2,3#] and Wei Tang [1,3#]

[1] State Key Laboratoty of Ecological Pest Control for Fujian and Taiwan Crops, College of Life Sciences , Fujian Agriculture and Forestry University, Fuzhou , 350002 , China

[2] Rice Research Institute, Fujian Academy of Agricultural Sciences ,Fuzhou 350018, China

[3] State Key Laboratory of Ecological Pest Control for Fujian and Taiwan Crops, College of Plant Protection, Fujian Agriculture and Forestry University, Fuzhou ,350002, China

[4] Fuzhou Institute of Oceanography, Minjiang University, Fuzhou ,350108

These authors contributed equally to this study

[#]Corresponding author emails: wangzh@fafu.edu.cn, tamgw@fafu.edu.cn , edwardhyh@163.com

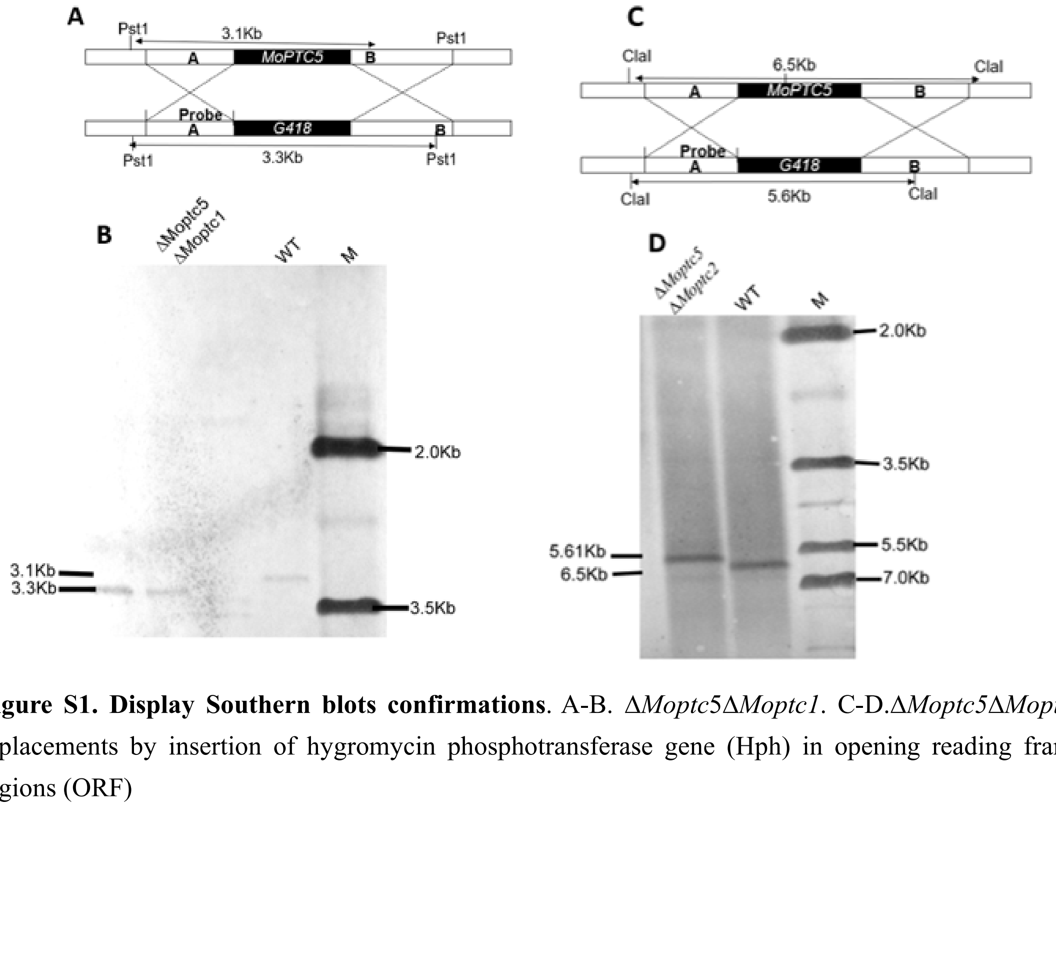

Figure S1. Display Southern blots confirmations. A-B. Δ*Moptc5*Δ*Moptc1*. C-D.Δ*Moptc5*Δ*Moptc2* replacements by insertion of hygromycin phosphotransferase gene (Hph) in opening reading frame regions (ORF)

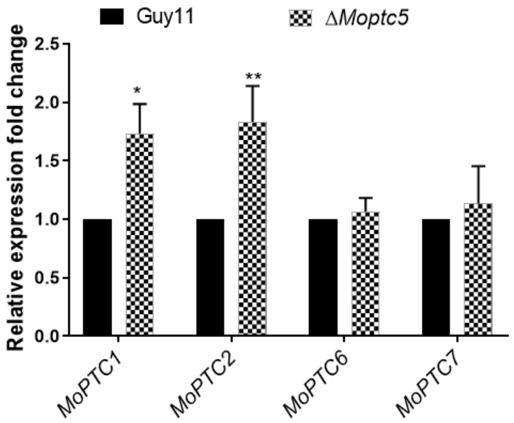

Figure S2. Transcripts expression profile of *MoPTC1, MoPTC2, MoPTC6* and *MoPTC7* in Δ*Moptc5* mutant . (β-actin gene was used as reference).

Table S1. Fungal strains (wild-type and mutants) employed during this study

Strain	Genotype description	Reference
Guy11	Wild-type	This study
Δ*Moptc5*	*MoPTC5* deleted mutant from Guy11	(1)
Δ*Moptc5*Δ*Moptc1*	*MoPTC1* deleted in the background of *MoPTC5*	This study
Δ*Moptc5* Δ*Moptc2*	*MoPTC2* deleted in the background of *MoPTC5*	This study
Moptc5_C	Complementation of *MoPTC5*	(1)

Table S2. List of primers used in this study

Primers	Sequence（5'-3'）	Application
MGG_MoPtc2 UA	CATCTTTCCGAGGTGGCG	Verification of replacement of ORF with hygromycin
MGG_MoPtc1 UA	CGGTCGGTGGCGGTAGTGAT	
MGG_ MoPtc2 AF	GCAATCTGAATCTCGTCCC	Amplification of A flagment
MGG_ MoPtc2 AR	GCCAAAATGAGATACCAGAC	
MGG_MoPtc1 BF	GCCAAAATGAGATACCAGAC	Amplification of B flagment
MGG_MoPtc1 BR		
MGG_ MoPtc2 OF	GCAATCTGAATCTCGTCCC	Verification of opening reading

MGG_ MoPtc2 OR	GGTGATGATGATGAGTTCT	flame (ORF)
MGG_MoPtc1 OF	TTCAACGACCACGAAAGC	
MGG_MoPtc1 OR	AAAACCACAGCCACTCCG	
MGG_ MoPtc2 AF	TTGTGATTCTGTCGGTTC	Amplification of A flagment
MGG_ MoPtc2AR	'GGAAATTGTAAGCGTTAATCTAGAGCGTTA CGGTTGACTCCTGAG	
MGG_ MoPtc2 BF	GCATTCTGGGTAAACGACTCATAGGAGTC CCCTACACCTTTGACCT	Amplification of B flagment
MGG_ MoPtc2 BR	GCATTCTGGGTAAACGACTCATAGGAGTC CCCTACACCTTTGACCT	
MoCHS1 QF	AGTGCGTTCGGATACGTTAG	Pcr primers for chitin sythases encoding genes
MoCHS1 QR	TGGTCACCGTGGAAGTATTG	
MoCHS2 QF	CGGTCCTCTTAGCCAGTATTTC	Pcr primers for chitin sythases encoding genes
MoCHS2 QR	TACGATCCTCAGCCAGATACA	
MoCHS3 QF	CAACGAGGACGAGGTTCTTT	
MoCHS3 QR	TCTTCTTCCATGCCTCCTTTC	Pcr primers for chitin sythases encoding genes
MoCHS4 QF	GAAGAGCTACGCGACAAGAA	
MoCHS4 QR	CTCGAAGCATTCAGCGATTTG	Pcr primers for chitin sythases encoding genes
MoCHS5 QF	GACTCTTGTTGGCGGTCTTTA	
MoCHS5 QR	CATCCCGGATACGAGCTAATTG	
MoCHS6 QF	CTTCGCTGGTGAGGTTGAATA	
MoCHS6 QF	GCCTACGATGTTGTTGAG	

Reference: Type 2C Protein Phosphatases MoPtc5 and MoPtc7 Are Crucialfor Multiple Stress Tolerance,Conidiogenesis and Pathogenesis of *Magnaporthe oryzae*
J. Fungi **2022**, 9, 1. https://doi.org/10.3390/jof9010001

Table S3. Physiological effects caused by deletion MoPtc5,MoPtc1 and MoPtc2 in *Magnaporthe oryzae*

Strains	Growth rate	Conidiation rate	Cell wall stresses	Osmotic stresses	Appressorium formation	Pathogenicity	Cell wall thickness	Appressorium turgor pressure
Δ*Moptc5*	Reduced	reduced	reduced	reduced	defected	reduced	Did not change	defected

Δ*Moptc5*Δ*Moptc1*	Highly reduced	Highly reduced	Significantly reduced	Highly reduced	Highly defected	Significantly reduced	Did not change	Highly defected
Δ*Moptc5*Δ*Moptc2*	Highly reduced	Highly reduced	Significantly reduced	Highly reduced	Highly defected	Significantly reduced	Did not change	Remarkably defected
